# Reference values for serum amyloid A, haptoglobin, lysozyme, zinc and iron in healthy lactating Lacaune sheep

**DOI:** 10.1186/s13028-018-0400-x

**Published:** 2018-08-06

**Authors:** Arianna Miglio, Livia Moscati, Emanuela Scoccia, Carmen Maresca, Maria Teresa Antognoni, Andrea Felici

**Affiliations:** 1 Department of Veterinary Medicine, Perugia University, Via S. Costanzo 4, 06126 Perugia, Italy; 2 Istituto Zooprofilattico Sperimentale dell’Umbria e delle Marche, Via Salvemini 5, 06126 Perugia, Italy

**Keywords:** Dairy ewes, Haptoglobin, Serum amyloid A, Serum trace elements, Sheep

## Abstract

Acute-phase proteins and trace elements are considered biomarkers of the immune response to infection, inflammation, trauma and other pathological conditions, as well as indicators of the health status and productivity of farm animals. Given the scarcity of published data on this topic, the purpose of this study was to determine the serum levels of serum amyloid A (SAA) and haptoglobin, as well as lysozyme, iron and zinc in clinically healthy Lacaune sheep in lactation months 3–7 ranging in age from 2 to 6 years. The mean serum levels for SAA (12.2 µg/mL), lysozyme (1.47 µg/mL), zinc (78.9 µg/dL) and iron (26.6 mmol/L) differed from those found in other sheep breeds and indicate considerable variations depending on breed, age and physiological status of the sheep. Reference values for clinically healthy mid-lactating Lacaune sheep were determined by using well-described and modern analytical and statistical methods. The reference intervals may be used to determine the health and welfare of lactating Lacaune sheep and may serve as a starting point to investigate diseases.

## Findings

Acute-phase proteins (APPs) are a group of proteins whose concentrations change significantly in the extracellular body fluids, e.g. as a consequence of inflammation, trauma or stress [1–4]. APPs are considered to be part of the nonspecific innate immune-response involved in the restoration of homeostasis and the restraint of microbial growth before acquired immunity is developed [5]. They are useful markers of inflammation in cows [3], but only a few studies have investigated APPs in sheep [6, 7]. The major APPs in ewes are serum amyloid A (SAA) and haptoglobin (Hp), whose serum concentrations are normally very low but increase markedly in response to acute and chronic challenges and increase moderately in subclinical inflammation [4–7]. APPs can also be used as indicators of health status in flocks and as prognostic indicators, because their levels are associated with disease severity.

Lysozyme is an enzyme that can damage bacterial cell walls by attacking peptidoglycan and is a biomarker of immune response. Its serum concentration serves as an indirect marker of inflammation, providing information about granulocyte activity and the functionality of the monocyte-macrophage system; in addition, it is a potential indicator of the amount of pathogens in the environment [8]. Few studies have investigated the serum levels of lysozyme in healthy sheep [8, 9].

The trace elements iron (Fe) and zinc (Zn) are necessary for the maintenance of normal metabolic states and productivity in animals. The blood levels of these elements are also indicators of immune function: their reduction is regarded as a nonspecific host-defense mechanism against bacterial infection; proinflammatory cytokines inhibit the export of Fe from the reticuloendothelial cells and affect ferritin synthesis and subsequent Fe storage. Moreover, during inflammation, an increase in the Zn-binding protein metallothionein has been documented [10–13]. Alterations in serum Fe and Zn concentrations have been reported in experimental coliform mastitis models in cows [10], but no studies in ewes have been reported.

The aim of this study was to determine the serum concentrations of SAA, Hp, lysozyme, Fe and Zn in clinically healthy mid-lactating Lacaune sheep.

The study was carried out at a dairy farm located in the Umbria region, central Italy. On eight different days during a 3-month period, 130 clinically healthy Lacaune sheep in lactation months 3–7 (2–6 years old) were selected. The sheep were examined clinically and standard hematology, biochemistry and serology was performed. Only sheep without a history of previous disease were selected. The sheep were fed mixed hay supplemented with cereal grains and were treated for endoparasites two times annually. Routine serological monitoring for small ruminant lentivirus infection was performed. An absence of overt signs of mastitis was confirmed as an absence of both abnormalities in the udder on inspection and palpation, and of macroscopic changes in mammary secretion. Blood samples were collected from the jugular vein into both EDTA and serum Vacutainer tubes. Moreover, milk samples from each udder half were subjected to bacteriological examination, and somatic cell counts (SCC) were determined to exclude sheep with subclinical mastitis (sheep with at least one milk sample with presence of mastitis bacteria and somatic cell count > 500,000 cells/mL) [7].

Sheep with a white blood cells count ≥ 15,000/μL were excluded. No abnormalities were detected in serum biochemistry. The concentrations of SAA and Hp were determined in serum samples by using specific commercially available ELISA kits (PHASE id RANGE, SAA and Hp Assay kit-Tridelta Development Ltd., Ireland) as previously described [14]. Serum lysozyme was assessed with lysoplate assays (Sigma-Aldrich, St Louis USA) [15]. Serum Fe and Zn levels were assessed with a Konelab 200 biochemical analyzer using specific kits (Sclavo Diagnostics, Italy).

Serum reference intervals (RIs) of each variable were determined through a nonparametric method, and 90% confidence intervals (CIs) were calculated (CLSI and QALS guidelines) [16]. The normality of distribution of the data was tested (Anderson**–**Darling test). Extreme outliers were detected through visual inspection of histograms and Tukey***’***s criterion, and were excluded from the calculation of RIs. Calculations were carried out in STATA 11.2 (StataCorp; Microsoft Excel 2003, Reference Value Advisor V2.1).

**Fig. 1:**
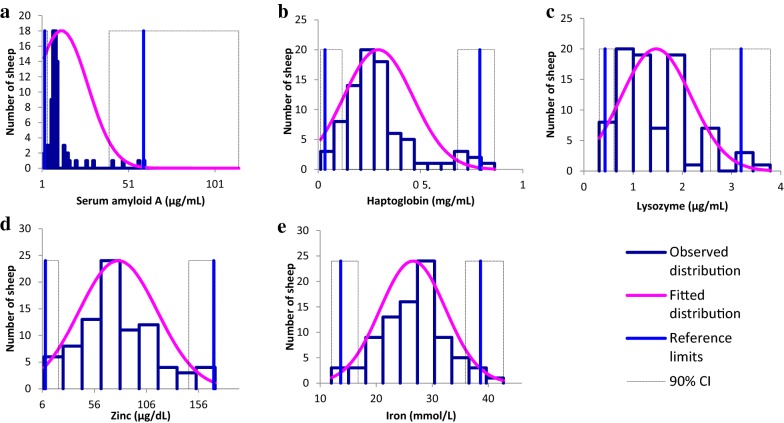
Observed (blue boxes) and fitted (purple lines) distributions of serum amyloid A (**a**), haptoglobin (**b**), lysozyme (**c**), zinc (**d**) and iron (**e**) from 86 clinically healthy mid-lactation Lacaune ewes; blue vertical lines are the reference limits with corresponding 90% confidence intervals as dotted lines

Twenty-two sheep were excluded due to leukocytosis and 22 due to subclinical mastitis leaving 86 sheep for inclusion. SAA, Hp and lysozyme were not normally distributed, whereas Zn and Fe were normally distributed. RIs and CIs are given in Table 1 and Fig. 1.

**Table 1:** Number of observations (Obs), median, minimum and maximum, lower and upper limits (90% confidence intervals, CI) of the determined reference intervals of ewes

Analyte		Obs	Mean	SD	Median	LRL 90% CI	URL 90% CI	Distribution	Method
Haptoglobin	mg/mL	83^a^	0.29	0.17	0.25	0.03 (0.01–0.12)	0.79 (0.68–0.86)	NG	NP
Serum amyloid A	µg/mL	85^b^	12.2	15.2	8.6	2.6 (2.1–4.2)	59.74 (39.8–114.8)	NG	NP
Lysozyme	µg/mL	85^c^	1.47	0.71	1.32	0.43 (0.31–0.61)	3.19 (2.57–3.78)	NG	NP
Zinc	µg/dL	85^d^	78.9	37.6	73.9	9.2 (7.8–21.7)	170.6 (146.7–171.9)	G	NP
Iron	mmol/L	86	26.6	5.8	26.9	13.7 (12–16.8)	38.6 (35.9–42.7)	G	NP

*LRL* lower reference limit, *URL* upper reference limit, *G* gaussian, *NG* non gaussian, *NP* non parametric

Lower letters excluded outliers: ^a^ 2.74, 2.18, and 2.98 mg/mL; ^b^ 239 µg/mL; ^c^ 4.81 µg/mL; ^d^ 259 and 254 µg/dL

The mean SAA level (12.2 μg/mL) was ten times higher than those (< 2 μg/mL) reported in recent studies in healthy Merino lambs [17] and non-lactating sheep [18, 19] but was within the range of those reported in healthy lactating Lacaune sheep (< 29.4 μg/mL) [18] and pregnant Scotch Mule sheep (17.9 μg/mL) [19] on the basis of the same assay.

In the literature, reported SAA levels in healthy animals vary considerably but remain below a cut-off value of 30 μg/mL [1, 17, 20, 21]. The upper limit of the 90% CI we found (2.6–59.74) is higher than this cut-off. Since SAA is a dynamic APP with a short half-life, SAA levels may be an early indicator of active ongoing inflammatory conditions in some animals included in this study. Neverthless, the absence of signs of disease in all ewe included may also suggest a breed-difference in SAA expression in Lacaune sheep, previously not reported. These results encurage further studies on the physiology of SAA in other breeds to standardize a cut-off value for healthy sheep and to detect possible breed differences.

The mean Hp level (0.29 mg/mL) was similar to published levels in healthy Merino, Scotch Mule and East Friesian sheep [17, 19, 22]. A Hp concentration above 1 mg/mL is considered the approximate cut-off of severe inflammation [17, 19]. In sheep, Hp increases locally through its release by neutrophils in response to early production of TNF-alpha in inflammatory reactions [20–24].

In veterinary medicine serum lysozyme levels differ among animal species, and breed-related differences have been reported [25]. In sheep, a high variability in lysozyme levels related to breed, age and sex have been reported [8, 9]. Breed-related differences are genetically determined: breeds homozygous for the primary gene encoding lysozyme have high lysozyme levels that would result in increased natural resistance against pathogens. Milk-type breeds have the highest lysozyme levels, with lower levels in lambs and higher levels in adults. The mean serum lysozyme level detected in this study (1.47 μg/mL) differs from those previously reported for lactating ewes [1, 20–24]. Particularly, we found a mean value higher than those identified in other milk-type breeds [8], which may indicate greater innate immunity in the Lacaune breed.

The mean serum values identified for Zn (78.9 µg/dL) and Fe (26.6 mmol/L) were different from those published in other lactating sheep [26]. Factors such as species, breed, sex, age, illness, seasonal and physiologic variations, nutritional content of the diet, feeding, environment and geography affect Zn and Fe serum levels [13, 26, 27]. We aimed at keeping these factors constant so that the RIs obtained would be more reliable but physiologic variations must be taken into consideration: serum Fe levels are lower in pregnancy and lactation, owing to the high demand for Fe in fetuses and neonates [13–16, 26]. The differences in analytical method, breed, sex and regional feeding practice may explain the discrepancy between our results and those previously published, because the physiological status was similar [9].

This is the first study to establish RIs for serum SAA, Hp, lysozyme, Zn and Fe in healthy mid-lactating Lacaune sheep, by using well-described and modern analytical and statistical methods. Published data often do not specify the number, age, breed, sex, geographic location or reproductive status of the sheep and the assay used to produce RIs [18, 22], thus making their use inaccurate for all types of sheep. The RIs identified could be used to assess the health and welfare of Lacaune sheep and may serve as a starting point to investigate diseases affecting this breed. Because a limitation of this study is the small sample size of animals included, gathering more data over time may improve RIs accuracy. Future studies should confirm the RIs for such variables in this species, because adequate published values regarding this are lacking.

## Abbreviations

APPs: acute-phase proteins
CI: confidence interval
EDTA: ethylenediamine tetra-acetic acid
Fe: iron
Hp: haptoglobin
Lys: lysozyme
RIs: reference intervals
SAA: serum amyloid A
SCC: somatic cell count
WBCs: white blood cells
Zn: zinc
